# Parameters Suggesting Spontaneous Passage of Stones from Common Bile Duct: A Retrospective Study

**DOI:** 10.1155/2019/5382708

**Published:** 2019-03-03

**Authors:** Tawfik Khoury, Mohamed Adileh, Ashraf Imam, Yosef Azraq, Avital Bilitzky-Kopit, Muhamad Massarwa, Ari Benson, Zaher Bahouth, Samir Abu-Gazaleh, Wisam Sbeit, Rifaat Safadi, Abed Khalaileh

**Affiliations:** ^1^Department of Gastroenterology and Liver Disease, Hadassah-Hebrew University Medical Center, Ein Kerem, Jerusalem, Israel; ^2^Department of Surgery, Hadassah-Hebrew University Medical Center, Ein Kerem, Jerusalem, Israel; ^3^Department of Radiology, Hadassah-Hebrew University Medical Center, Ein Kerem, Jerusalem, Israel; ^4^Department of Surgery, Kaplan Medical Center, Rehovot, Israel; ^5^Institute of Gastroenterology and Liver Diseases, Galilee Medical Center, Bar Ilan Faculty of Medicine, Israel

## Abstract

**Background:**

Common bile duct (CBD) stones are common. However, they are known to pass spontaneously, which obviates the need for ERCP.

**Aim:**

The aim of this study is to identify specific predictors for spontaneous passage of CBD stones.

**Methods:**

Data was retrospectively collected for all patients who were hospitalized with clinical, laboratory, or ultrasonographic evidence of choledocholithiasis and who underwent magnetic resonance cholangiopancreatography (MRCP) in Hadassah Medical Center between 2005 and 2011. The patients were classified into 4 groups: group A (positive MRCP and positive ERCP), group B (positive MRCP but negative ERCP), group C (positive MRCP but did not undergo ERCP), and group D (negative MRCP that did not undergo ERCP) for choledocholithiasis. All positive MRCP-groups (A+B+C) were further grouped together into group E. We compared groups A versus B and groups E versus D.

**Results:**

Comparing groups A versus B, only gamma-glutamyl transferase predicted spontaneous passage of stones from CBD, as the level was significantly higher in group A (677±12.1) versus group B (362.4±216.2) (P=0.023). Patients with small stone diameter (P=0.001), distal stones (P=0.05), and absence of intrahepatic dilatation (P=0.047) tend to pass their stones spontaneously. Comparing groups D versus E, it was found that male gender (P=0.03), older age (P<0.001), high levels of GGT (P=0.022), high levels of alkaline phosphatase (P=0.011), high levels of total bilirubin (P=0.007), and lower levels of amylase (P<0.001) are predictors for positive MRCP studies for CBD stones.

**Conclusion:**

Identification of specific predictors is important to avoid unnecessary invasive endoscopic intervention.

## 1. Introduction

Gallstone disease is a commonly encountered disease worldwide. The incidence increases with age and females are twice as likely to be affected as compared to male patients [1]. Common bile duct (CBD) stones are seen in approximately 3-15% of patients with gallbladder stones [2, 3]. CBD stones can either be clinically asymptomatic and only manifested by elevated cholestatic liver enzymes and bilirubin or present with more severe disease such as ascending cholangitis [4]. The gold standard test for diagnosis and treatment of CBD stones is endoscopic retrograde cholangiopancreatography (ERCP), which has 100% specificity [5]. However, ERCP is associated with complications, including post-ERCP pancreatitis, cholangitis, retroperitoneal perforation, postsphincterotomy bleeding and anesthesia related complications [6]. Thus, ERCP should be performed only when clinically indicated. Magnetic resonance cholangiopancreatography (MRCP) has emerged and replaced ERCP for diagnosis of CBD stones as it identifies up to 91% of biliary stones, but still stones smaller than 5 mm were detected only in 71% of cases [7]. In some patients diagnosed with CBD stones on MRCP, the subsequent ERCP does not detect CBD stones. These findings suggest that the CBD stones had probably passed spontaneously prior to ERCP. Thus, the endoscopic management strategy of CBD stones has focused mainly on timing as there is an accumulating evidence which shows that CBD stones often pass spontaneously without the need for endoscopic or surgical drainage procedures [8, 9]. However, there is a scarcity of data regarding specific factors that predict such spontaneous passage.

The aim of the present study is to examine clinical and radiological MRCP parameters that predict spontaneous CBD stone passage.

## 2. Study Design and Methods

We retrospectively collected data of all MRCPs performed at Hadassah University Medical Center between July 2005 and February 2011. Of the 3,412 MRCPs retrieved, we excluded all cases with primary liver or bile duct disease (including cirrhosis, primary sclerosing cholangitis, hepatitis), and we also excluded all liver transplant and hepatobiliary oncological patients. Patients were enrolled if they were suspected to have CBD stones and met the following inclusion criteria: clinical presentation consistent with biliary colic, obstructive jaundice, ascending cholangitis, or pancreatitis in addition to elevated cholestatic liver enzymes and suspected gallbladder stones after evaluation with ultrasonography.

**Figure 1:**
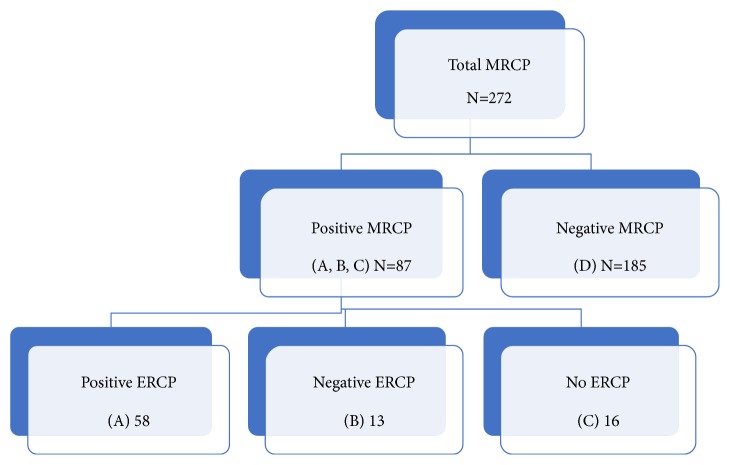
Flow chart of the study cohort.

MRCP was considered positive if stones were diagnosed within the CBD. Patients with a positive MRCP were then further divided into two groups: those who underwent ERCP and those who did not undergo subsequent ERCP. Positive ERCP was defined as the presence of bile duct stones which were seen as filling defects on fluoroscopic examination. Patients were divided into 4 groups. Group A included patients with positive MRCP and positive ERCP for bile duct stones. Group B included patients with positive MRCP but negative ERCP for bile duct stones. Group C included patients with positive MRCP but did not undergo ERCP and group D included patients with negative MRCP and also did not had ERCP. All positive MRCP-groups (A+B+C) were also combined to form an additional group, group E (Figure 1). To evaluate potential eligibility criteria to perform MRCP following initial suspension of CBD stones, we compared group E (A, B, and C) to group D. Moreover, to examine the predictive parameters for spontaneous bile stone passage, we compared group A with group B. The study was approved by the institutional local IRB committee number 0316-12-HMO.

## 3. Data Collection

All data (demographic features, laboratory results, and imaging files) were analyzed. MRCP studies were revised by senior radiologist with greater than ten years of experience reading hepatobiliary imaging and who were blinded to the clinical course and outcome of the patients. Variables that were documented on MRCP included stone size, site (proximal and distal; distal stones were defined as stones impacted in the distal third of the CBD), number of stones, and common bile duct dilatation.

## 4. Study Endpoints

The primary endpoint of the study was to characterize specific factors that predict spontaneous passage of CBD stones among laboratory (aspartate transaminase (AST), alanine aminotransferase (ALT), lactate dehydrogenase (LDH), white blood count (WBC), gamma-glutamyl transferase (GGT), and alkaline phosphatase (ALKP)) and radiological variables. All laboratory tests that were included in the analysis were collected near the timing of MRCP performance, mostly up to one day before MRCP. The secondary endpoint was to examine eligibility variables to proceed to MRCP.

## 5. Statistical Analysis

Data was reported as mean ± standard deviation for quantitative variables and frequencies (percentages) for categorical variables. To assess the differences between groups A and B (positive MRCP + (positive versus negative ERCP)) and between groups D and E (positive versus negative MRCP) we used independent samples T-test or Mann-Whitney* U* test for quantitative variables and *χ*2-test or Fisher's exact test for categorical data. All p values were two-sided and statistical significance was set at P< 0.05. Statistically significant variables were tested through ROC curve analysis to determine a threshold above which an ERCP test is recommended. The data was analyzed using IBM SPSS version 21.

## 6. Results

### 6.1. Demographics and Clinical Characteristics

Overall, 272 patients were included in the final analysis. Group A included 58 patients (mean age 65.3±18.9 years, 33 males (56.9%)). Group B included 13 patients (mean age 68.4±18.9 years, 8 males (61.5%)). Group C included 16 patients (mean age 64.6±19.5 years, 6 males (37.5%)), and group D included 185 cases (mean age 55±20.4 years, 74 males (40%)). Most patients had one CBD stone (53.1%), while the prevalence of having 2, 3, 4, 5, and 6 stones was 23.4%, 10.9%, 6.3%, 4.7%, and 1.6%, respectively.  Table 1 shows the clinical characteristics comparing groups A and B (positive MRCP + (positive versus negative ERCP)). There was no difference in age, gender, length of hospitalization, and temperature at admission in group A as compared to group B (P=NS). However, the patients in group A underwent MRCP earlier compared to patients in group B (4.8±2.5 and 6.6±2.6 days, respectively; P=0.008).  Table 2 shows the same clinical parameters comparing groups E and D (positive versus negative MRCP). The patients in group D were significantly younger (55±20.4 years) compared to group E patients (65.6±18.9 years) (P<0.001). Male gender percentage was significantly higher in group E (54%) as compared to group D (40%) (P=0.03). The median hospitalization days of group E patients were significantly higher than that of group D patients (P<0.001).

### 6.2. Laboratory Findings

Table 3 shows the laboratory results of groups A versus B. Only GGT predicted spontaneous passage of stones from CBD, as its level was significantly higher in group A (677±12.1) as compared to group B ((362.4±216.2), P=0.023). However, no difference was seen in other laboratory parameters. Similarly, Table 4 displays the laboratory results of groups E (all MRCP positive tests) and D (negative MRCP). GGT, alkaline phosphatase (ALK), and total bilirubin were significantly higher in group E as compared to group D (601.4±468.5, 322.2±235.1, and 57.6±49.5 versus 445.3±390.8, 235.8±213.1, and 40.6±34.1; P = 0.022, 0.011, and 0.007, respectively), while the level of amylase was significantly lower in group E as compared to group D (118.7±211.8 versus 653.6±1293.7, respectively, P<0.001). The rest of the laboratory findings were similar among the two groups.

### 6.3. MRCP Radiological Findings of the Study Groups

To assess radiological criteria in patients who tend to spontaneously pass their stones, we compared groups A and B (Table 5). We observed that a lower percentage of intrahepatic bile duct dilatation (P=0.047), smaller stone diameter (P=0.001), and distal bile duct stones (P=0.05) was the radiological predictors of stone passage. There were no differences in the number of stones and common bile duct diameter when comparing the two groups.

### 6.4. Receiver Operating Characteristics (ROC) Curve Analysis

A ROC analysis was used to define a threshold for stone diameter and GGT levels above which an ERCP exam is indicated. The threshold was defined for the value with maximal sensitivity and specificity. Regarding stone size, according to our results a stone larger than 3.5mm has low chance to pass spontaneously. This cut-off has sensitivity of 71%, specificity of 69%, positive predictive value (PPV) of 35%, and negative predictive value (NPV) of 91%. For GGT the cut-off was defined as 408 IU/L with a sensitivity of 68%, specificity of 61%, PPV of 32%, and NPV of 89%.

## 7. Discussion

Spontaneous CBD stone passage has been reported by few studies. A recent study reported that more than half of patients with obstructive jaundice had spontaneous passage of the gallstones from the CBD [10]. Our study was designed to examine the clinical, laboratory, and radiological factors that might predict spontaneous passage of CBD stones. We compared characteristics of patients who had a positive MRCP and subsequent positive ERCP to those of patients with a positive MRCP and subsequent negative ERCP and we also compared patients with a positive MRCP (notwithstanding results of subsequent intervention and testing) with those who had a negative MRCP. These comparisons allowed us to identify factors related to spontaneous passage of CBD stones either from the time of MRCP until ERCP or from the time of the initial suspension of CBD stones until MRCP performance.

The results of our study revealed that advanced age was a risk factor for failure of spontaneous passage of CBD stones. A previous study also showed that advanced age is a risk factor for cholelithiasis [11] which increases the chance of CBD stones, yet this study showed that female gender is associated with an increased risk of cholelithiasis and subsequent CBD stones, while our results showed that male gender was associated with failure of spontaneous passage of CBD stones. Few previous studies reported spontaneous passage of CBD stones after MRCP alone [12, 13]. In our study, only improvement in GGT level predicted spontaneous CBD stone passage when comparing groups A versus B, while GGT, ALKP, and total bilirubin predicted stone passage when comparing groups E versus D. In a previous study, it was shown that GGT has the highest predictive value and diagnostic accuracy in predicting common bile duct stones [14]. Another study reported GGT, ALKP, and total bilirubin as a sensitive test for choledocholithiasis; however, of them, GGT was the most sensitive marker that was abnormal in 92% of CBD stones cases [15]. Furthermore, we found that intrahepatic bile duct dilatation, stone diameter, and distal bile duct stones were radiological predictors of stone passage. Conversely, there were no differences in the number of stones and common bile duct diameter among groups.

Additionally, we generated ROC curve analysis for size of stone and GGT level and found that stone size of less than 3.5 mm is more likely to pass spontaneously with acceptable sensitivity (71%) and specificity (69%). A previous study has shown that CBD stones less than 5 mm in diameter were more likely to spontaneously pass without the need for further intervention [16] and another study reported an increased rate of stone retention in the CBD with a larger stone size [17]. To the best of our knowledge, there are no previous studies that reported that distal CBD location is a predictive factor for spontaneous stone passage as we showed in our study. Moreover, the presence of intrahepatic dilatation suggests continuous CBD obstruction and hence is an indirect marker for retained CBD stone.

Our study has several limitations. It was a retrospective study which increases the potential for bias of data collection. In addition, the study was performed in a single medical center and the study groups were not similar in number of patients included. Nonetheless, the main strength of our study was that we included a relatively large cohort of patients with definite diagnosis of biliary stones after exclusion of other inflammatory or malignant conditions.

In conclusion, spontaneous passage of CBD stones is common. Identification of specific laboratory and radiological markers may aid in clinical decision-making regarding subsequent invasive endoscopic intervention. Further randomized clinical trials are warranted to establish predictors of spontaneous CBD stones passage.

## Figures and Tables

**Table 1:** Clinical characteristics of the study population: groups A and B.

Clinical parameter	Group A	Group B	P value
Age (years; mean ± SD)	65.3±18.9	68.4±18.9	0.55
Male gender % (N)	56.9 (33)	61.5 (8)	0.76
Length of hospitalization (days; median, range)	15 (4-62)	16 (11-50)	0.612
MRCP day (number of days from admission; mean ± SD)	4.8±2.5	6.6±2.6	0.008
Temperature at admission (mean ± SD)	36.8±0.7	36.4±0.5	0.056

**Table 2:** Clinical characteristics of the study population: groups D and E.

Clinical parameter	Group E	Group D	P value
Age (years; mean ± SD)	65.6±18.9	55.0±20.4	<0.001
Male gender % (N)	54 (47)	40 (74)	0.03
Length of hospitalization (days, median, range)	15 (4-83)	9 (4-43)	<0.001
MRCP day (number of days from admission; mean ± SD)	5.1±2.5	5.5±2.7	0.304
Temperature at admission (mean ± SD)	36.8±0.7	36.8±0.6	0.907

**Table 3:** Comparison of blood tests at admission: groups A and B.

Blood test	Group A	Group B	P value
Creatinine	100.4±88.7	106.8±63.6	0.68
AST	337.7±263.2	336.2±420.9	0.278
ALT	311.3±237.2	246±333.7	0.088
LDH	1081.6±803.4	1150.1±1079.8	0.87
WBC	9.8±3.3	10.1±2.8	0.494
Platelets	235.1±85.3	227.7±88.7	0.677
GGT	677.0±512.1	362.4±216.2	0.023
ALKP	345.1±256.4	214.5±90.5	0.078
Amylase	126.8±244.1	69.8±38.4	0.806
Bilirubin	57.5±35.9	41.0±34.6	0.092

**Table 4:** Comparison of blood tests at admission: groups E and D.

Blood test	Group E	Group D	P value
Creatinine	99.7±79.7	85.3±49.5	0.137
AST	334.2±295.4	285.8±236.4	0.250
ALT	299.4±269.7	269.2±249.1	0.444
LDH	1066.9±816.3	1000.1±773.6	0.604
WBC	9.9±3.3	10.3±4.0	0.446
Platelets	234.2±83.6	257.1±100.4	0.104
GGT	601.4±468.5	445.3±390.8	0.022
ALKP	322.2±235.1	235.8±213.1	0.011
Amylase	118.7±211.8	653.6±1293.7	<0.001
Bilirubin	57.6±49.5	40.6±34.1	0.007

**Table 5:** Radiological predictors of spontaneous CBD stone passage.

Radiological marker	Group A	Group B	P value
Stone size (mm)	5.88±3.3	3.12±1.1	0.001
Intrahepatic dilation (%)	53.8	23.1	0.047
Distal stones (%)	63.5	92.3	0.05
CBD diameter (cm)	1.01±0.34	0.85±0.38	0.36
Number of CBD stones	1.92±1.28	1.77±1.16	0.64
Cholelithiasis (%)	65.4	83	0.31

## Data Availability

The data are found in the hepatobiliary unit in Hadassah Medical Center.
